# Anonymising interview data: challenges and compromise in practice

**DOI:** 10.1177/1468794114550439

**Published:** 2015-10

**Authors:** Benjamin Saunders, Jenny Kitzinger, Celia Kitzinger

**Affiliations:** Keele University, UK; Cardiff University, UK; University of York, UK

**Keywords:** anonymity, coma, confidentiality, minimally conscious, research ethics, serious brain injury, vegetative

## Abstract

Anonymising qualitative research data can be challenging, especially in highly sensitive contexts such as catastrophic brain injury and end-of-life decision-making. Using examples from in-depth interviews with family members of people in vegetative and minimally conscious states, this article discusses the issues we faced in trying to maximise participant anonymity alongside maintaining the integrity of our data. We discuss how we developed elaborate, context-sensitive strategies to try to preserve the richness of the interview material wherever possible while also protecting participants. This discussion of the practical and ethical details of anonymising is designed to add to the largely theoretical literature on this topic and to be of illustrative use to other researchers confronting similar dilemmas.

## Introduction

This article addresses the practical and ethical challenges faced when anonymising qualitative interview data. We draw on examples from our interview study with more than 50 individuals who have a relative with a chronic disorder of consciousness, i.e. who are in a vegetative or minimally conscious state with little or no awareness of themselves or their environment. This is a highly sensitive and contentious area – especially when it comes to decision-making about serious medical treatments, which was one of the key areas addressed in our research. We examine the issues that arose as we attempted to anonymise these data, and we provide empirical examples that invite readers to consider what choices they would make if faced with similar issues. It is through this focus on *practice* that the original contribution of this article will be made. The aim is to complement the growing theoretical literature on anonymisation (which often debates the issues at a mainly abstract level) and to be of practical use to other researchers encountering similar challenges.

### Defining anonymity

In much previous literature ‘anonymity’ has commonly been used either interchangeably with, or conflated with, ‘confidentiality’ (e.g. Kaiser, 2009; Tolich, 2004). For us, ‘confidentiality’ is a generic term that refers to *all* information that is kept hidden from everyone except the primary research team. Anonymity is one form of confidentiality – that of keeping participants’ identities secret. However, confidentiality also includes keeping private what is said by the participants, something only achievable through researchers choosing not to share parts of the data.

An idealised view of anonymity is that a person will never be traceable from the data presented about them. As we will show, however, guaranteeing complete anonymity to participants can be an ‘unachievable goal’ in qualitative research (Van den Hoonaard, 2003: 141). Some commentators argue that since the primary researchers know who participants are, true anonymity is by definition never achievable, i.e. there will always be at least one person with access to participant information (Scott, 2005: 247). In most contexts, however, in-depth qualitative research could not be carried out without breaching anonymity so defined: researchers not only know participants’ identities, but usually meet them in person. For this reason we would argue that it rarely makes sense to define ‘anonymity’ in the research context so broadly: the primary researchers will always be in the know. In what follows we address the question of anonymity insofar as it applies to persons *other than* the primary researchers.

### Anonymising data: a balancing act

The process of anonymisation is a complex, and far from water-tight process, in which changing people’s names or disguising locations are only first steps in a more nuanced process around managing ‘identifying details’. Anonymity is a continuum (from fully anonymous to very nearly identifiable) (Scott, 2005: 249), along which researchers balance two competing priorities: maximising protection of participants’ identities and maintaining the value and integrity of the data.

Many official ethics guidelines recommend disguising the personal identities of research participants as a *default* position – see, for example, the Statement of Ethical Practice for the British Sociological Association (BSA, 2002), and the Economic and Social Research Council’s Framework for Research Ethics (ESRC, 2012). This represents the ‘normalisation’ of anonymity (Tilley and Woodthorpe, 2011: 198). Others recommend respecting the wishes of participants either for anonymity or to ‘receive recognition’ (Canadian Sociological Association (CSA), 2012: s.13, Statement of Professional Ethics) and anthropologists, in particular, have long been concerned about informants’ requests to be properly credited (Cassell and Jacobs, 1987). The ethics of ‘anonymity by default’ is challenged by critics who champion the empowering effect that they claim can be fostered by participant identification (Giordano et al., 2007; Grinyer, 2002) and argue against paternalism and an exaggeration of the proposed ‘harm’ that may result from lack of anonymity (Moore, 2012). Others highlight the negative effects that default anonymisation can have on research outcomes (e.g. Walford, 2005: 2), arguing, for example, that keeping places and settings hidden ‘naturalizes the decoupling of events from historically and geographically specific locations’ (Nespor, 2000: 549), or that anonymisation can leave underlying power structures unchallenged, preventing the pursuit of transformative research goals (Baez, 2002).

There are also practical challenges. Concealing identities can sometimes be virtually impossible (Van den Hoonaard, 2003). Anyone closely tied to a particular research setting will likely be able to recognise participants and places (Nespor, 2000; Scheper-Hughes, 2000), what Tolich (2004) refers to as threats to ‘internal confidentiality’; and since academics tend to use geographically convenient locations this makes research sites more easily traceable (Walford, 2005). However, commentators also point out that the difficulties faced in anonymising do not justify its abandonment (Kelly, 2009), that many research participants still wish their identities to be concealed (e.g. Corden and Sainsbury, 2006) and that they often do not have a clear idea about how their words will be used, such that the future harm from naming participants cannot always be reliably predicted (Wiles et al., 2012: 47).

The debate summarised above highlights the need for a contextually-contingent approach to anonymising data. Those writers who have defended the principle of anonymity as default do not necessarily equate this with ‘blanket’ anonymisation, i.e. disguising every single identifying detail mentioned, which corresponds with the advice of most ethics guidelines, e.g. Qualidata (the specialist ESRC qualitative data archiving service) and the British Sociological Association guidelines (BSA, 2002: 5). However, these bodies give little advice about how to anonymise in practice; and with the exception of a few detailed accounts of challenges in particular projects (e.g. Clark, 2006; Clough and Conigrave, 2008; Kaiser, 2009), this gap is also evident in the broader academic literature debating anonymisation issues. This article contributes toward filling this gap.

### Research context and method: challenges for anonymisation

Our research explored family experiences of having a relative in a prolonged disorder of consciousness – either a vegetative state (VS) where the patient is diagnosed as having no awareness of self or environment, or a minimally conscious state (MCS) in which the patient has only minimal and intermittent awareness. The second and third authors are the Principal Investigators (PIs) on the project. The first author is the Research Associate whose responsibility it was to anonymise the transcripts and upload them into a data management programme which was shared with a larger interdisciplinary research group across two universities. The group’s publications draw on our interview analysis to show how families of severely brain injured individuals experience decision-making about serious medical treatments (Kitzinger J and Kitzinger C, 2013; Kitzinger C and Kitzinger J, 2014), how they understand ‘consciousness’ (Nettleton et al., 2014), construct ‘death’ (Holland et al., 2014) and engage with the law (Halliday et al., 2014).

Anonymisation in this project was particularly important. Participants shared very sensitive and personal information, not only about themselves, but also about third parties. The patients they are talking about are profoundly incapacitated, and our interviewees revealed intimate details about their conditions and treatments. Many also discussed end-of-life decision-making in relation to their relative, and sometimes said things that they explicitly stated they had never (and *would* never) voice to anyone else (e.g. that they wished their relative had died). With this in mind, we assured participants that we would endeavour to keep their identities hidden as far as we could through changing their names and the names of anyone else they mention (e.g. treating clinicians), as well as disguising place names and particular identifying details. However, participants were also made aware of the limits to the anonymity we could offer, as well as the challenges that can be posed to maintaining anonymity, as discussed throughout the rest of this article.

Anonymisation in this project was particularly challenging. First, the two PIs who designed the research, recruited participants and conducted all the interviews (Jenny Kitzinger and Celia Kitzinger) have ‘insider’ status: their sister, Polly Kitzinger, was severely brain injured in a car crash in 2009. As other researchers have noted, insider research is associated with a set of particular benefits and dilemmas (e.g. Breen, 2007; Taylor, 2011); it clearly helped with recruitment and increased participants’ willingness to share their experiences, and to do so very openly, but it also posed challenges for anonymity. In some interviews, participants drew on shared ‘insider’ knowledge about particular healthcare settings, and healthcare professionals. This makes their identities potentially traceable, since the identities of the PIs, and their brain-injured sister, is public knowledge (Kitzinger C and Kitzinger J, 2011). Second, there are relatively few patients in the UK with chronic disorders of consciousness. This ‘small population’ problem has been discussed in relation to *ethnographic* studies (Van den Hoonaard, 2003; Walford, 2005), which often focus on a particular village or town, where there is a high risk that individuals may recognise themselves in the talk of others (e.g. Hopkins, 1993, or see problems encountered in relation to Carolyn Ellis’ account of *Fisher Folk* (1986) discussed in Ellis, 1995). However, similar problems can arise with a geographically dispersed population with unusual characteristics. We recruited from across the UK but some families had already met each other – or we learned that they had done so subsequent to our interviews – in specialist rehabilitation settings (of which there are very few across the UK), or through charities and support groups, including online forums. Most were also in contact with the same small set of experts (e.g. experts in assessing consciousness or in related legal issues).

The insularity of our research context posed a threat to what Tolich (2004) terms ‘*internal*’ confidentiality – raising the possibility of participants identifying themselves, or other members of their own family or from other families with whom they have had contact. This possibility was increased by our use of ‘snowball sampling’, including deliberately pursuing interviews with different members of the same family – some of whom did not want parts of what they said to be identifiable to other family members.

There was also a risk to anonymity in relation to what Tolich (2004) refers to as ‘*external*’ confidentiality. Tolich’s use of the term confidentiality relates to the ‘protection against identification’ (2004: 101), thus equating with our use of the term ‘anonymity’. Our participants might be identified by some members of the intended *audience* for our research findings – which includes the leading medical, scientific and legal experts in the field. The small number of PVS/MCS patients means that an idiosyncratic detail such as a quoted vocalisation from a patient (e.g. calling out a particular word while apparently in a vegetative state) could identify that patient and his/her family to the medical or legal professionals involved. A reference to participation in scientific trials might be similarly revealing – a pertinent issue as we deliberately sought to interview families with experience of the novel neurotechnology, fMRI (see Samuel and Kitzinger J, 2013). Likewise, we sought out interviews with those rare families involved in legal cases for withdrawal of artificial nutrition and hydration (Kitzinger C and Kitzinger J, 2014), of which there have been fewer than 100 cases in total in England and Wales since 1993 to the present.

A further complication is the issue of the ‘small world’ (or ‘global village’) established via the world wide web – in which what one might know within a family, community, service user or professional network becomes potentially available to anyone with online access. Our capacity to ensure anonymity was compromised by the availability of transcripts of court cases, coroners’ reports and media coverage related to some families, as well as participants’ own use of social media to campaign (e.g. to raise funds for treatment) (see also Wiles et al., 2012; Saunders et al., 2014).

In the following sections we will discuss how we attempted to tackle the challenges that arose and provide examples of what we did in relation to the following factors:

1. People’s names
2. Places
3. Religious or cultural background
4. Occupation
5. Family relationships
6. Other potentially identifying information

In selecting illustrative examples of where anonymity was particularly problematic we have to ensure that we do not breach the very anonymity we have tried to protect. The examples presented in this article are all drawn from our already anonymised dataset. Because we cannot (without breaching anonymity) compare what was actually said with our anonymised version of it, we have in some cases developed a second anonymised version of the same data especially for this article.

## Anonymising: six key areas

### People’s names

The most common form of anonymisation discussed in the literature (see, for instance, Clark, 2006: 5; Moore, 2012: 332) consists of assigning pseudonyms. Some research participants wanted us to use their own names and that of the patient. They felt that their relative had been stripped of their identity/voice first by the brain injury and then by a system which ignored their prior expressed values and beliefs and silenced those attempting to represent them (similar passionate wishes to be identified have been reported by other researchers, e.g. one mother saw the use of pseudonyms as a betrayal of her dead son’s memory: Grinyer, 2002: 3). Despite this, we reluctantly took the decision to avoid naming interviewees wherever this could be negotiated – especially since at times there were also legal issues (e.g. where a court required that the identity of a patient should be protected). Naming some participants might reveal the identities of people they talked about (Clark, 2006) and might also compromise the anonymity of other interviewees (Kelly, 2009).

In choosing pseudonyms, we wanted to avoid revealing too much about the ethnic/cultural backgrounds of participants (see ‘Religious and cultural backgrounds’ section, below), but we did select names that would at least in some way resonate with them (Grinyer, 2002: 3). Of course another option would have been to avoid pseudonyms altogether – especially given that other studies suggest that some participants may prefer simply to be referred to by characteristics of gender and age-range (see Corden and Sainsbury, 2006). This seemed to us, however, to be quite impersonal and also would make it harder for readers to follow individual narratives. (For an account of why we opted for names rather than numbers, see Kitzinger J and Samuel, 2014: 6–7.)

On occasion, however, anonymising required us to prevent readers from recognising that two data extracts were spoken by the same person (e.g. when neither extract on its own threatened anonymity but might in combination make identification possible). Then we sometimes did avoid using a pseudonym for one of the extracts or went further and created a smoke screen by attributing different pseudonyms to each extract. (For other examples of this smoke screen strategy, see Kaiser, 2009). This is clearly a compromise in relation to the integrity of the data, and could mislead readers, but we judged it necessary when (for example) interviewees expressed concerns about endangering relationships with clinicians to whom they referred using derogatory nicknames (e.g. ‘Mr God Almighty’) or when they described certain contentious activities such as smuggling pets into the hospital, taking illegal recreational drugs into the care home, or reading their relative’s medical notes when they were not entitled to do so. Interestingly some interviewees seemed quite fluent in strategies for anonymising – perhaps because some had become familiar with managing parallel identities on the internet. For example, one woman suggested changing pseudonyms for her, as she reflected on some highly personal information about the impact of her husband’s brain injury on her own life:

Extract 1

The thing is me and John [husband] had quite a – *obviously if you use this part I would change the [pseudonymous] names* [our emphasis] *–* we had a very dominating relationship […] I was very overpowered by John. And I had no say in anything I did […]. The music I listened to, the TV shows I watched, everything was very controlled. […] Even walking to the bus stop you know, [he’d say] ‘I’m coming with you’, but I knew it was just to check I wasn’t running off with somebody else. It was very, very restrictive. […] He wasn’t violent towards me, but he’d be the kind that would throw a wardrobe next to me.

This interviewee had fought tirelessly against some clinicians’ suggestions that further life-sustaining treatment for her husband might be futile, and she had found her own voice in the process.

Extract 1 (continued)

I have to say I found liberation through John’s tragedy. *And whilst I’ve been very careful who I say that to, it’s the truth*. You know, through what’s happened to him I’ve found myself. […] So it’s been empowering in his disempowerment. And it’s really horrible to say that, but it’s true.

Given the sensitive nature of these comments we followed the interviewee’s own suggestions, using a pseudonym for this extract which is different from the pseudonym used for other quotes from her. In addition, we edited out specific identifying details (e.g. that she has now joined a dance class, something which her husband had previously forbidden [a fictionalised example]).

In other cases, it was *interviewer*, rather than interviewee, who initiated discussion of the option of omitting a pseudonym or using multiple pseudonyms. For example, after one mother described an occasion when she thought about killing her severely brain injured son (not, in fact, an unusual experience for our interviewees, see Kitzinger J and Kitzinger C, 2013):

Extract 2

I thought ‘if I loved him as much as I say I do, I would not permit this [suffering]’. […] And I stood there looking at him […] thinking ‘I could do this. I could. I know exactly what to do. All I’ve got to do is put my thumb over his trachy [tracheostomy, pipe into his throat] and his pillow over his face. It’d be done in five minutes.’

At the end of this interview, the *researcher* suggested it might be appropriate to take extra precautions to ensure that this episode was anonymised, and the interviewee agreed (‘I don’t think I’ve actually – well [two family members] know about that, but I haven’t told anybody else’). Although she wanted her anonymity protected, this interviewee actively wanted to have this part of her interview used:

… to illustrate the point that if there is not a safe option [to allow a dignified death] then people – when they’re overwhelmed with tiredness, grief, desperation and hopelessness – will put the boundaries aside just to do something to ease an unbearable situation.

As well as ‘ring-fencing’ particularly sensitive extracts, using two pseudonyms for the same person also enabled us to include some potentially identifiable aspect of a narrative in some circumstances. For example, in an article focusing on participants’ engagement with the law (Halliday et al., 2014), we included data from two families in which the patient had been killed by another member of the family (both involving subsequent legal proceedings that received media coverage). In this one article we used pseudonyms different from those used in our other publications to reduce the likelihood that any identification made possible by our use of these particular extracts would compromise the anonymity of extracts from the same individual used in other articles.

### Places

Our assignment of ‘place’ pseudonyms followed the same principles outlined above, and we commonly replaced identifying places with numbers (e.g. ‘Hospital 1’, ‘Hospital 2’) or generalised descriptions (e.g. ‘Southampton’ became ‘South Eastern English city’). Such anonymisation represents yet another compromise to the integrity of the data as anonymising places can result in decontextualisation, limiting the scope for analysis (Baez, 2002; Nespor, 2000). However, the small number of healthcare institutions specialising in treating serious brain injury means that naming these, or even the town/city in which they are located, could lead to the identification of particular families, as well as the clinicians who worked with them. We did, however, ensure that settings were not entirely decontextualised by differentiating between England/Wales and the Republic of Ireland, for instance (and between countries inside and outside the EU), since these distinctions might be relevant due to transnational variations in legal framework and healthcare policies. A reference to a patient’s injury having occurred while skiing in Bulgaria, for example, may be replaced with ‘sporting accident’ in ‘Overseas, EU country no.1’, and so forth. For our own analytic purposes, we also kept track of every individual care setting mentioned to inform a forthcoming publication about how people talk about particular settings, such as a modern neurorehabilitation unit or the Victorian hospital building, thereby addressing some of Nespor’s (2000: 549) concerns about the ‘decoupling of events from historically and geographically specific locations’. For this particular publication exploring talk about settings, we plan to cross-link locations but to remove or change pseudonyms, allowing a discussion of ‘place’ without revealing which of our interviewees was linked to which location.

### Religious and cultural backgrounds

References to interviewees’ religious, cultural or ethnic background could result in ‘deductive disclosure’ (Kaiser, 2009: 1632) compromising anonymity. Following the advice of ethical guidelines, such as those offered by Qualidata (UK Data Service, 2013), we often replaced religion/culture with similar but unrelated items, or with generalised descriptions (e.g. Jaspal’s Hinduism became ‘Jaspal’s religious faith’).

This strategy became problematic when religion, culture or ethnic identity was not simply ‘background information’ but crucial ‘context for deeper and fuller understanding’ (Clark, 2006: 6). After lengthy discussion we decided to retain certain features in some cases. For example George’s brother, David, had been injured six years prior to the interview, and at the time was diagnosed by medical professionals as having ‘low awareness’. George and his wife, Linda, (interviewed together) were very optimistic about David’s situation. Their religion underpinned their shared views about David’s future, along with a strong sense of family togetherness that they associated with their cultural heritage (from outside the UK). They consistently represented these factors as underpinning their belief in David’s ongoing recovery, as illustrated in the following extract:

Extract 3

Interviewer: You have very strong family values. That comes through.

George: Yeah.

Linda: Mm.

Int: Do you have religious beliefs as well?

George: Oh yeah, we’re [specified faith].

Linda: Yes, yes.

George: My [relative’s] a [religious leader]. So, yeah.

Int: And does this influence your approach, do you think?

Linda: Ah, saying about the [place of worship] now. We take

David to the [place of worship] as well. And the first day we

took David to [place of worship] […] I gave him the

candle, David, and he went like this [miming clenching hand around candle] […] The other time when we lit a candle and gave it to him, he was holding it like that. And he was holding it for a while and then he would go like that [miming hand going lax].

Although it was the interviewer who initially raised the topic of religious faith, the joint narrative that George and Linda subsequently constructed clearly indicates the importance of religion in their lives and they went on to explain at length how David interacted with other artefacts crucial to their religion and how this informed their belief that David had more understanding than the doctors had predicted. They saw David’s actions as clear evidence that he was able to recall the significance of religious objects, and thereby to connect with the beliefs he held prior to his accident – such that an important part of his identity had survived the devastating brain injury.

In partially anonymising this example by removing reference to any specific religion (although even ‘candles’ are only relevant in some religions and not others) we tried to do justice to the story that George and Linda were telling without being too specific. We suspected that, so strong were their religious and cultural values, George and Linda would not have been comfortable if we had represented them as belonging to a different religious faith or culture. We compromised through cutting references which would narrow down identification of their religion/cultural background too tightly – this involved losing some very interesting data but seemed a reasonable compromise, albeit one which made the quotations much more anodyne than they had been in the original form. The question we ask ourselves here is whether we have veered too far, and ‘white-washed’ the data, ‘forfeiting much of the richness yielded by the study’ (Parry and Mauthner, 2004: 144) and draining it of meaning?

### Occupation

Like religious and cultural beliefs, occupation too could be highly salient to our interviewees and therefore difficult to simply remove or alter. Where possible we again followed the advice of ethical guidelines, such as those offered by Qualidata, by substituting general terms, e.g. Jaspal’s job as a theatre director became ‘job in the Arts’. However, sometimes such vagueness was incompatible with conveying key parts of a story. For example, Sandra, whose daughter has been in PVS for many years, describes the ‘tipping point’ for her, which triggered a discussion about how her daughter might be allowed to die:

Extract 4

I’m a beauty therapist by trade so I looked after her hands and toenails. And on this occasion one of the nursing staff […], decided her nails were a bit long and had cut them and in one case had gone across the quick and it had bled. And it had really upset me because I know how much that hurts. And anyway, [X] came in and she said ‘You look really rough’. And I said ‘You know, there are times, [X], when I just wish she would go to sleep and never wake up’. And she said ‘Well if that’s what you truly, truly want, it can be achieved’. And I sort of looked at her and said ‘Oh yes? And how?’ And she said ‘Seriously. There is a process.’ […] So after she’d gone, I actually sat down and thought about it, consulted with my [other family members] who basically said ‘Oh, thank god!’

Sandra isolates a precise moment which, for her, encapsulated her daughter’s suffering and her own inability to protect her; it is through the lens of her professional experience that this moment gains its significance. Anonymising Sandra’s occupation would undermine her description of this important moment, and therefore risk not fully representing Sandra’s experiences.

### Family relationships

The familial relationship between the interviewee and the severely brain injured patient is usually highly significant for the interviewee (e.g. ‘As his mother I know …’ or ‘Speaking as her husband …’). Wherever possible we retained the family relationship in our published accounts – but we sometimes felt it necessary to disguise them. We have, for example, usually not identified gay relationships as such and we have subsumed categories such as ‘step-parent’ or ‘adopted child’ under the category ‘parent/child’ to avoid identification. We recognise the problems associated with this. In particular, the attempt to achieve anonymity structurally predisposes researchers toward the erasure of the experiences of minorities – precisely because their minority status may make these research participants easy to identify as, for example, the only known lesbian with breast cancer in a medical practice (Kaiser, 2009) or one of very few black faculty members (Baez, 2002). The result is a one-dimensional view which obscures family diversity. With regret, we pursued this strategy nonetheless, except when the particular nature of the minority relationship was essential to our analysis (e.g. if an interviewee compared being in the role of ‘step-parent’ versus ‘his “real” mother’, or an interviewee commented on discrimination encountered as a gay partner).

When publishing extracts that reveal potentially identifying minority or atypical relationships we have made sure that such extracts are presented in isolation from other data from the same person and/or discussed with interviewees the issues involved and arrived at a negotiated decision about how to use the data. One such example comes from the interview with Louisa, whose two teenage sons, Nicholas and Dylan, were involved in an accident which left Dylan with relatively minor injuries, but Nicholas in a vegetative state. The anonymisation challenge here centred on the fact that Louisa’s sons were identical twins. The likelihood of there being more than one mother in the UK with identical twins, one in PVS, is small. We initially decided that it would be easy enough to substitute any reference to ‘twin’ with ‘brother’. This proved problematic, however, when faced with extracts such as the following:

Extract 5

Interviewer: Did you have a sense of where was Nicholas’ soul or spirit … through all this?

Louisa: Yes. I think I know where it is.

Int: Where?

Louisa: That photograph is absolutely Nicholas [pointing to photograph of Nicholas before his accident]. And I think Nicholas’ soul is settled with his brother. I’ve got a photograph on my iPad where Dylan looks like the 23-year-old version of that. Now I mean you would think ‘Well of course he would, you silly woman! They’re identical twins!’. But I could always tell who was who because of the way they held their faces. Because Dylan’s view of the world was different to Nicholas’, so they always had slightly different expressions … And that became more apparent as they grew up. But this picture that I’ve got of Dylan the day he graduated, he looks like his bro. And I can’t help thinking that because they were identical twins […]. I just wonder if Nicholas is now just living out his destiny through his brother’s life. […] So I suspect that maybe that spirit that split in the early part of [date of accident] has now merged again. I hope so. I hope so.

As with Extracts 2–4, this part of the interview gives important insights into how an interviewee thinks about life–death issues in relation to a severely brain injured relative. It could not have been included in our analysis if we had not retained ‘twins’. Towards the end of the interview, the interviewer asked Louisa for her input on our anonymising strategy:

Extract 6

I’m not really too worried about whether people hook up on the fact that it’s us or not […] There aren’t too many families who [have these unique traits] so frankly, if anybody’s remotely interested, they’ll realise it was the Herwell family. […] Just take your normal steps [to anonymise the interview], but don’t worry.

This kind of researcher–participant collaboration on an individualised basis has been suggested by others (e.g. see Grinyer, 2002; Kaiser, 2009; Svalastog and Eriksson, 2010) but none have raised the possibility of discussing these issues during the interview itself, as was done here – this strategy does, however, rely on the interviewer to recognise challenges to anonymity as the interview progresses.

### Other potentially identifying issues

Over and above name, place, occupation and religion there are often features in any dataset, which may provide unique (or closely narrowing) identifying information. We have given examples of a range of such features throughout the discussion above – including idiosyncratic details of a case. Depending on the substantive focus of the research there may also be a recurring issue that needs special attention. In our case this was the nature of the original injury. Such information was often interwoven throughout accounts and also has prognostic significance. Brain damage resulting from ‘non-traumatic’ injury (e.g. from oxygen deprivation following cardiac arrest) is more predictably devastating that that resulting from traumatic brain injury (e.g. from a blow to the head). Distinguishing between these types of injury is therefore analytically important. However, beyond this we commonly used generic phrases such as ‘road traffic accident’ or ‘sporting injury’, which allowed us to describe a range of events while not revealing more specific information.

So far, so straightforward. However, sometimes the cause of the injury had a greater significance to the interviewee and was infused throughout the account. For instance, precipitating events, such as domestic violence or a cardiac arrest brought on by anorexia could be hard to simply evacuate from an interviewee’s story. There may be times that unpicking such elements in the narrative would be core to the analysis, and therefore justifiably retained in published reports, as long as the participant was assured of being kept anonymous as far as this is possible. Suppose, for example, that an interviewee told us that it was horrific to consider withdrawing artificial nutrition and hydration (ANH) from her PVS daughter because that daughter had suffered from anorexia and the whole relationship between mother and daughter was embedded in this very fraught relationship with food. Further suppose that this case had come to court, and a transcript was available on the internet referring to the daughter’s anorexia. This is sufficiently unusual (in fact there is no such case!) that it would identify not only the mother and daughter but also other members of the family and any health professionals mentioned in the interview. In anonymising this interview we might perhaps change the daughter’s illness from anorexia to oesophageal cancer – another illness (albeit physical rather than psychological) causing problems in eating which might therefore similarly affect how the mother felt about ANH withdrawal. If we think that disguise may be too transparent, the daughter may become a son, and/or the mother transformed into a sister, and we would use different pseudonyms for different extracts to minimise the chance of jigsaw identification.

## Conclusion

We have shown that anonymising is not, in practice, something that can be done on automatic pilot with a *‘*one size fits all’ or ‘find and replace’ approach, and have highlighted some of the challenges we faced in one particular dataset. For us, anonymising has been an ongoing working compromise: at times we have sacrificed some of the integrity of the data in order to maximise anonymity and at other times we may have risked compromising anonymity in order to maintain the integrity of the data. The process of anonymising transcripts has involved multiple meetings and lengthy email exchanges between the three authors of this paper: it has been very much a team effort, with not all of us agreeing all of the time. We share the process in the hope it can help others debate the issues and try to come to the ‘best possible’, or ‘least worst’, option for their own data.

We would like to conclude by highlighting the implications of this discussion for the design and conduct of research more broadly. First, our work highlights some of the considerations that might help to maximise ‘informed consent’ via the design of consent forms, information for participants and dialogue during the research process. Our consent form (approved by both universities’ ethics committees) included from the outset a range of permissions, from giving consent for only the project team to access the data through to archiving via Qualidata. This evolved over the course of the study as we expanded the range of ways in which we aimed to use the data and wanted to alert participants to this and to give them a more nuanced set of options from which they could opt in or out. For example, as we were increasingly invited to talk at medical ethics conferences we started to highlight the fact that the likely audience for participant data would include treating clinicians, and possibly the very clinicians who were currently, had previously, or might in future, treat the interviewee’s relative (a point also explored by Kaiser, 2009). In addition, during the course of the project, we became more skilled at detecting, during the course of an interview, possible problems with anonymising what interviewees were saying, and were able to raise it with participants there and then (as shown in Extract 6). In other cases we contacted a participant subsequently to discuss particular concerns. Experience also taught us that we needed to alert participants to the possibility of *future*, currently unanticipated, risks to their anonymity. If, for example, after giving us permission to use their interview in our research, they were subsequently to use the same vivid descriptions in (for example) a court case or on their blog, this could allow readers to make a connection. This too is something we now explicitly include in our information sheet for participants.

Our research also has implications for data sharing and archiving. To manage data sharing we followed a two-stage process: first a crude anonymisation of whole transcripts so that we could enter them into the data management system to share with our research group, and then anonymising individual data extracts at the point of submission for publication, leaving in only details essential to the point being made, and creating a smoke screen where necessary (e.g. gender change). Even the first level of anonymising was extremely time-consuming – and therefore has resource implications for the funding of data sharing. The first author (as research associate) anonymised names of people and places and any other obvious identifiers, but left in information which was either difficult to extract because it infused the whole interview, or which was essential for the analysis. More than a month’s labour was devoted to anonymising just 20 of the interviews (three week’s full-time from the first author with additional input from the two co-authors) and we ran out of resources to anonymise all the data we had permission to share. Costing this into future funding proposals needs to recognise the time commitment involved. As we worked on anonymising transcripts it also became increasingly apparent how much would be lost if we created maximum protection for our participants and how difficult it would be to make full transcripts completely anonymous for use by other researchers in an academic archive. In collaborating with colleagues from different disciplines we have become acutely aware that there are different assumptions across disciplines about what constitutes appropriate anonymisation and varying levels of experience in handling sensitive empirical data. It is difficult for colleagues detached from the original data collection context, and without in-depth knowledge of the field, to judge what may, or may not pose an identification risk. If we archive the interviews after we have finished working with them and permit their use by other researchers, we will have to do another round of anonymising and proceed with great care as our ongoing oversight of anonymity issues in relation to our dataset has been an essential part of our current work with our co-authors.

Finally, we have to admit that, even with all our efforts, anonymity cannot be completely guaranteed. It can be subverted from different directions, not least because of the major challenges to anonymity linked to the rapid changes we are seeing in the data available on the world wide web, and associated practices (see Saunders et al., 2014; Tilley and Woodthorpe, 2011). Particular challenges are posed especially through the increasing use of social media (both by research participants and researchers) and open access publishing of academic work (a condition of some funding) and increasing efforts at public engagement from academia (e.g. via impact activities). For example, our latest work involves developing a ‘healthtalkonline.org’ website for families and clinicians – where they can learn about families’ experiences from the research, and hear actual audio, or view vivid extracts of film, from interviews with some individuals. This initiative is funded by an ESRC ‘Knowledge Exchange’ grant, and is an invaluable addition to the existing research and its outputs: ensuring the research findings are presented in an accessible and engaging way that can support families in the future, be used in clinical training to improve practice and, perhaps, impact on policy. However, inevitably such initiatives raise new challenges for how to negotiate anonymity (or its suspension) with family members who chose to appear on film (even if they do so with pseudonyms). In such cases the sort of careful thought that goes into anonymising practices outlined in this article is useful groundwork for considering how to manage deliberate suspensions of anonymity (e.g. through the use of voice recordings or actual film of the individual on the site). Explicit negotiation with research participants is also inevitably an essential part of the process (e.g. participants sign off on how they wish to be described in a mini biography on the site as well as being offered view of their transcripts or copy of the film). New practices also have to be used under certain circumstances; for instance, in one case a participant was happy to have her voice used, but not her image, and requested that her voice be disguised a little on the recording.

Discussion between researchers and research participants thus becomes an essential part of negotiating levels of anonymising, or its suspension under particular circumstances – a process which is valuable, but time consuming, and again needs to be costed into projects adopting such approaches. It also needs to be recognised that although researchers have professional duties and training in relation to anonymity, research participants may not, and sometimes research participants may take the initiative to disrupt anonymity or adopt ‘identity management’ approaches in unexpected ways. As we were writing this article, we were confronted with one such challenge: one of our research participants was googling information about coma and came across our (open access) article about brain injury which drew on our, oh so carefully and fully anonymised, interview data (Kitzinger J and Kitzinger C, 2013). He cheerfully emailed to inform us that he had worked out his own pseudonym, and linked the article to his *Facebook* page. This, to us, seemed an ironic twist, and one that almost typifies the difficulties in anonymising our dataset. We no longer set the terms of engagement as researchers, and anonymising is very much an evolving exercise that continues to throw up challenges and surprises – and this is subject to further reflection in Saunders et al. (2014), which also reproduces a revision we made to our consent form to manage similar situations in future.
